# Behavioral risk assessment of exposure to wild and domestic animals in response to a Marburg virus disease outbreak, Ghana 2022

**DOI:** 10.1016/j.onehlt.2025.101010

**Published:** 2025-03-14

**Authors:** Richard Suu-Ire, Shannon Ball, Meyir Yiryele Ziekah, Jean DeMarco, Morgan Kain, Amos Sarpong Agyei, Jonathan H. Epstein

**Affiliations:** a The University of Ghana, Accra, Ghana; b EcoHealth Alliance, NY, New York, USA; c Forestry Commission of Ghana, Kumasi, Ghana; d One Health Science, NY, USA

**Keywords:** Marburg virus disease, Risk assessment, Zoonotic spillover, Infectious disease outbreaks, Ghana

## Abstract

In July 2022, Ghana reported its first outbreak of Marburg virus disease (MVD). The source of the outbreak was unknown. In August 2022 we conducted a behavioral risk assessment, surveying 715 participants in three rural communities associated with the presumptive index case: Site 1 in Ashanti Region and Sites 2 and 3 in the Western Region of Ghana. Our primary aim was to characterize exposure to wild and domestic animals, specifically Egyptian rousette bats (ERBs), the natural reservoir for Marburg virus. We focused on two primary routes of potential exposure to ERBs: 1) eating fruit bearing bite marks and 2) entering caves or mines where bats were present. Eating fruit bearing bite marks was common across all sites, but highest at Site 2 in the Western Region. Higher levels of education were negatively correlated with eating fruit bearing bite marks, while having fruit trees present on the participant's home compound increased the odds of this exposure. Residents in Site 3 were significantly more likely to be exposed to bats in caves and mines. Participants across all sites also reported high levels of exposure to bats inside buildings; while ERBs do not typically roost in buildings, this presents a potential risk of exposure to other bat-associated pathogens. One participant at Site 3 reported symptoms consistent with MVD in the previous four months, suggesting the possibility of unrecognized cases that may have been associated with the outbreak. This study identified behaviors within the outbreak regions that could increase the risk of exposure to Marburg virus and other bat-borne pathogens. Serological surveys in these communities would provide important information about the extent of the Marburg outbreak by identifying unreported cases, as well as exposure to other filoviruses.

## Highlights

• We administered a risk assessment to 715 people in three communities impacted by Ghana's 2022 Marburg virus disease outbreak.
• We identified opportunities for potential exposure to Marburg virus and other bat-borne pathogens.
• We specifically analyzed factors associated with potential exposure to Egyptian Rousette bats, a known Marburg reservoir.
• Key exposures included eating fruit bearing bite marks and entering caves/mines where bats were present.

## Graphical abstract

**Figure:**
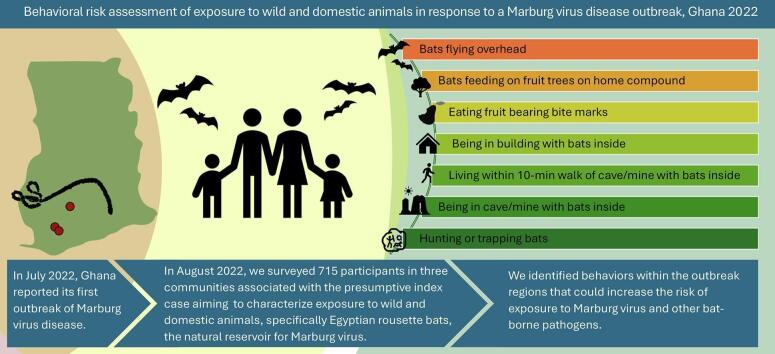

## 1 Introduction

In July 2022, Ghana reported its first confirmed case of Marburg Virus Disease (MVD) [1]. The 26-year-old male had been living and working on a farm in the Western Region of Ghana, before falling ill in late June and relocating to join his family in the Ashanti Region. He was hospitalized on June 26 and died the following day. He tested positive for MVD on July 1 at the Noguchi Memorial Institute for Medical Research, and the results were confirmed by the Institute Pasteur, Dakar in Senegal [[1], [2], [3]]. Two additional cases from the same household – a child who died in hospital and an adult female who survived – were confirmed later in July [2]. No other contacts tested positive, and the Ghanaian Ministry of Health (MoH) declared the outbreak over on September 16, 2022 [1].

Marburg virus (MARV) is the causative agent for MVD and a member of the filovirus family. MARV causes severe viral hemorrhagic fever (VHF) with an average case fatality rate of 50 % [[4], [5], [6], [7]]. Symptoms include fever, severe headache and malaise, muscle aches, abdominal pain, nausea, vomiting, diarrhea, rash, and bleeding from multiple areas, and it is transmitted *via* direct contact with infected people or materials contaminated by their fluids [5]. Egyptian Rousette bats (*Rousettus aegyptiacus*), or ERBs, are a natural reservoir for MARV [[8], [9], [10]]. ERBs are fruit bats (family *Pteropodidae*) that typically roost in caves or mines and feed on fruit [5,11]. Previous MVD outbreaks, including the 2024 outbreak in Rwanda, have been linked to entering caves or mines [5,12]. MARV has also been shown to persist on fruit eaten by ERBs, presenting a plausible route of spillover to humans and other primates [13]. Some domestic animals, including pigs and dogs, are susceptible to filoviruses, though their role in transmission to humans is poorly understood [[14], [15], [16], [17]]. Recently, MVD outbreaks have increased in frequency and geographic range, with outbreaks also reported for the first time in Guinea in 2021, Equatorial Guinea and Tanzania in 2023, and Rwanda in 2024 [[18], [19], [20]].

In support of the Ghanaian MoH's response to the 2022 MVD outbreak, EcoHealth Alliance and the University of Ghana (UG) conducted a behavioral risk assessment in communities associated with the presumed index case to identify potential routes of MARV exposure. While the presumed index case may have been infected by another, unidentified human case, he was considered most likely to have been infected by an animal. As such, our primary objective was to characterize contact with wild and domestic animals, focusing on exposure to known MARV hosts (specifically, ERBs) and their environments. We also collected data describing community demographics, household characteristics, history of illness, travel patterns, and health practices and beliefs.

## 2 Methods

### 2.1 Study design

**Fig. 1:**
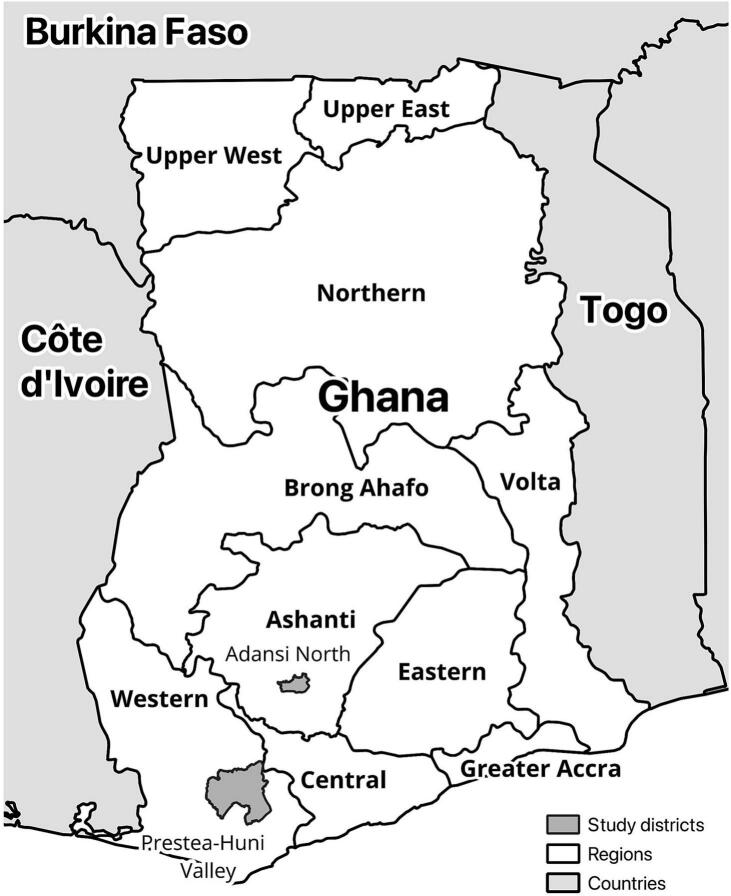
Map of study districts, Ghana.

For this cross-sectional study, we visited three communities where the presumed index case had spent time prior to his death: Site 1 in the Adansi North District of the Ashanti Region, and Sites 2 and 3 immediately neighboring each other in the Prestea-Huni Valley District of the Western Region (Fig. 1). At the time of our site selection, immediately following the outbreak, it was not yet known where the index case had fallen ill. Later, it was established that the index case had been living and working on a farm in Site 3 at the time of symptom onset, after which he relocated to join his family at Site 1. Despite this, we analyzed surveys from all sites to gain broader insight into potential filovirus spillover risk in different areas of rural Ghana. Data collection was conducted between August 25 and September 26, 2022.

### 2.2 Questionnaire

An electronic questionnaire was developed using Open Data Kit (ODK) [21] and administered on Samsung Galaxy tablets running Android operating systems (Android version 11 One UI version 3.1). We based our questions on previous studies of zoonotic disease spillover risk, collecting information on participant demographics, household characteristics, and health beliefs and behaviors, as well as animal exposure, travel patterns, and symptom history within the four months prior to interview. Four months was considered a reasonable recall period, included the index case's infectious period (June 2022), and covered approximately four MARV incubation periods (21–28 days) [22]. As assessing symptom history was not our primary objective, we did not ask about specific episodes of illness. Instead, we asked if participants had experienced individual symptoms or combinations of fever with other key symptoms, based on the World Health Organization's MVD case definition, at any point in the previous four months. Key symptoms included severe headache and fatigue; severe nausea, vomiting, or diarrhea; rash; or bleeding or bruising unrelated to injury [5].

### 2.3 Sampling and sample size calculation

Target sample sizes were calculated to ensure that a sufficient number of participants was selected to represent the population at each site. We used the below formula for sample size calculations with finite populations, where n is the sample size, z is the z-score, $\hat{p}$ is the population proportion, E is the margin of error, and N is the population size. Based on available population estimates for each site, 95 % confidence intervals, 5 % margins of error, and estimated population proportions of 50 %, we obtained target sample sizes of 323 in Site 1, 318 in Site 2, and 347 in Site 3.

$$n^{\prime }=\frac{n}{1+\frac{z^{2}*\hat{p}\left(1-\hat{p}\right)}{{ℇ}^{2}*N}}$$

Sampling was conducted in two stages. First, households were selected by haphazard sampling, though with broad geographic coverage of each site. Fully random selection of households was not feasible as we did not have access to detailed maps or current census data, and flooding and a lack of roads prohibited access to some areas of Site 3. Second, if the head of household agreed, we used ballots to randomly select one individual from all present household members aged 12 years or older.

### 2.4 Data collection

Participants provided verbal informed consent. Enumerators asked questions in the local language and recorded responses in ODK forms on electronic tablets. Field team leaders completed observational site characterization reports.

### 2.5 Data analysis

Data were analyzed in R version 4.2.2. Summary tables stratified by site were produced to describe population demographics (Table 2), household characteristics (**Supplement 1**), medical history and health beliefs (Table 3, **Supplements 2–3)**, travel (**Supplements 4–8**), and animal exposures (Table 4, **Supplements 9–11**).

We used multivariable logistic regression models to examine risk factors associated with two key types of potential ERB exposures: 1) eating fruit bearing bite marks, and 2) having been in a cave or mine in the past 4 months where bats were present. For each model, we included variables identified *a priori* that were assumed to have causal associations with the potential ERB exposure. When assessing eating fruit bearing bite marks, variables of interest included in the model were site, gender, age, highest level of education, and the participant having fruit trees present on their home compound. For bat exposure in a cave or mine, variables of interest included were site, gender, age, highest level of education, and reporting mining as a livelihood. In both models, site was included to account for any un-measured variation by location.

Age was analyzed as a continuous variable and centered for interpretability. For the model of recent bat exposure in a cave or mine, we restricted our analysis to Sites 2 and 3, as only one participant at Site 1 reported this exposure. Fewer than 10 participants at Sites 2 and 3 reported college/university/professional as their highest level of education; thus, we combined this category with secondary school. Collinearity between independent variables was examined using variance inflation factors and no collinearity was detected in any models. The logistic regression results are expressed as odds ratios with 95 % confidence intervals.

### 2.6 Ethics statement

Institutional Review Board approval was not obtained, as this was considered public health practice as part of the official outbreak response [23]. We received an official letter of permission for our study from the Ghana Health Service, and at each site we met with local authorities, including regional and/or district health directors and traditional community leaders, to obtain community-level permission.

## 3 Results

We administered 715 surveys in total, including 318 at Site 1 in the Ashanti Region, and 135 and 262 at neighboring Sites 2 and 3 in the Western Region, respectively. While the surviving adult female was not available, our survey does include some family members and close contacts of the MVD cases.

### 3.1 Site characterization

All three sites were rural with low human density, and each had close proximity to natural areas. We observed bats flying overhead (species unknown) and roosting inside houses and other buildings at all three sites. Pig farming was not common at any site, although one pig pen containing >20 pigs was observed at Site 1, and there were reports that small-scale pig farming may be increasing at Site 3 (Table 1). Upon visiting the small farm at Site 3 where the index case had been living and working, we observed maize, yams, cassavas, and fruit trees growing on site, as well as disturbed rat burrows indicative of rodents on the compound.

**Table 1:** Site characteristics.

	**Site 1**	**Site 2**	**Site 3**
Region	Ashanti	Western	Western
District	Adansi North	Prestea-Huni Valley	Prestea-Huni Valley
Estimated population	1830	684	3573
Estimated number of households	452	182	511
Human density	Rural	Rural	Rural
Estimated number of pigs present at site	20–100	<20	<20

### 3.2 Demographics

The majority of participants were female in Site 1 (63 %) but male in Site 3 (58 %), and even numbers of males and females were sampled in Site 2 (50 % each). Approximately 80 % of participants at Sites 1 and 2 reported their highest level of education as primary school or above, compared to 59 % at Site 2. Crop production was the most common livelihood across all sites. 16 % of Site 3 participants reported working in mining (Table 2).

**Table 2:** Participant demographics.

	Ashanti Region		Western Region					
	Site 1 (*n* = 318)		Site 2 (*n* = 135)		Site 3 (*n* = 262)		Total (*n* = 715)	
Variable	n	%	n	%	n	%	n	%
**Gender**								
Male	119	37.4	68	50.4	151	57.6	338	47.3
Female	199	62.6	67	49.6	111	42.4	377	52.7
**Age**								
12–19	47	14.8	17	12.6	34	13.0	98	13.7
20–29	70	22.0	34	25.2	76	29.0	180	25.2
30–39	63	19.8	25	18.5	60	22.9	148	20.7
40–49	35	11.0	17	12.6	42	16.0	94	13.1
50–59	32	10.1	15	11.1	27	10.3	74	10.3
60–69	41	12.9	20	14.8	16	6.1	77	10.8
70–79	19	6.0	4	3.0	4	1.5	27	3.8
80+	10	3.1	3	2.2	0	0.0	13	1.8
**Highest level of education**								
None/prefer not to say	62	19.5	27	20.0	109	41.6	198	27.7
Primary school	165	51.9	82	60.7	117	44.7	364	50.9
Secondary school	68	21.4	19	14.1	34	13.0	121	16.9
College/university/professional	23	7.2	7	5.2	2	0.8	32	4.5
**Place of worship**⁎								
Church	284	89.3	106	78.5	236	90.1	626	87.6
Traditional	4	1.3	1	0.7	4	1.5	9	1.3
Mosque	25	7.9	19	14.1	9	3.4	53	7.4
None	6	1.9	10	7.4	13	5.0	29	4.1
**Livelihood**⁎								
Animal farming/production	39	12.3	4	3.0	26	9.9	69	9.7
Crop planting/production	171	53.8	102	75.6	232	88.5	505	70.6
Animal trade/market business	20	6.3	5	3.7	7	2.7	32	4.5
Meat processing, slaughterhouse, abattoir	1	0.3	0	0.0	0	0.0	1	0.1
Housework/childcare	18	5.7	5	3.7	4	1.5	27	3.8
Student	48	15.1	12	8.9	15	5.7	75	10.5
Construction	12	3.8	3	2.2	2	0.8	17	2.4
Migrant laborer	17	5.3	36	26.7	102	38.9	155	21.7
Mining	7	2.2	10	7.4	43	16.4	60	8.4
Hunter/trapper	3	0.9	12	8.9	19	7.3	34	4.8
Fisher	0	0.0	0	0.0	1	0.4	1	0.1
Bat guano collection	0	0.0	0	0.0	0	0.0	0	0.0
Veterinary/animal care	0	0.0	0	0.0	0	0.0	0	0.0
Forest product collector	4	1.3	1	0.7	7	2.7	12	1.7
Nurse, doctor, community health worker	8	2.5	0	0.0	0	0.0	8	1.1
Other	124	39.0	46	34.1	41	15.6	211	29.5
Prefer not to say	1	0.3	2	1.5	0	0.0	3	0.4

*Note:* Percentages calculated by site total.

⁎ Select all that apply.

### 3.3 Medical history

111 (16 %) participants reported experiencing severe, acute illness that lasted >2–3 days within the previous four months (**Supplement 1**). Of those 111, 74 (67 %) reported fever in combination with at least one other symptom associated with MVD at some point in the four-month period (Table 3**, Supplement 3**). One Site 3 participant reported experiencing fever with bleeding or bruising unrelated to injury, as well as fever with severe headache and fatigue and fever with severe nausea, vomiting, or diarrhea within the previous four months. Five participants at Site 1 reported fever with rash in the previous four months. All five also reported fever with severe nausea, vomiting, or diarrhea, and three also reported fever with severe headache and fatigue.

**Table 3:** Potential MVD symptoms among participants who self-reported fever within the prior 4 months.

	Ashanti Region		Western Region					
	Site 1 (*n* = 52)		Site 2 (*n* = 21)		Site 3 (*n* = 38)		Total (*n* = 111)	
Symptoms⁎	n	%	n	%	n	%	n	%
Fever with severe headache and fatigue	29	55.8	12	57.1	28	73.7	69	9.7
Fever with severe nausea, vomiting, or diarrhea	16	30.8	3	14.3	9	23.7	28	3.9
Fever with rash	5	9.6	0	0.0	0	0.0	5	0.7
Fever with bleeding or bruising not related to injury	0	0.0	0	0.0	1	2.6	1	0.1

*Note:* Percentages calculated by total participants self-reporting fever by site.

⁎ Select all that apply.

### 3.4 Animal exposures

Participants across all three sites commonly reported raising/caring for dogs or having them come inside their household. Swine contact was reported by less than 2 % of participants. Other animals that participants reported exposure to included rodents, goats/sheep, cats, and other animals, which mainly included poultry. The most common contact types reported were raising/caring for animals (70 %) or having animals come inside the house (67 %). Hunting or trapping animals was rarely reported, except for hunting rodents, which was most common in Site 2 (23 %, **Supplements 9–10**).

Being inside a building with bats was one of the most common bat exposures across all three sites, reported by 23 % of participants. 22 % of participants in Site 3 specifically reported bats inside their house, compared to 9 % in Site 2 and 11 % in Site 1. 27 % of participants also reported bats feeding on fruit trees on their home compound. Approximately 45 % of participants in Sites 2 and 3 and 28 % in Site 1 also reported eating fruit bearing bite marks. In Site 2, 18 % of participants reported exposure to bats inside a cave or mine in the previous four months, compared to only 6 % in Site 3 and 0.3 % in Site 1. Fewer than 4 % of participants across all sites reported hunting/trapping, slaughtering/cooking, or eating bats (Table 4).

**Table 4:** Participant-reported direct and indirect bat exposures.

	**Ashanti Region**		**Western Region**					
	**Site 1 (n** **=** **318)**		**Site 2 (n** **=** **135)**		**Site 3 (n** **=** **262)**		**Total (n** **=** **715)**	
**Exposure type**⁎	**n**	**%**	**n**	**%**	**n**	**%**	**n**	**%**
Bats seen flying overhead in last 4 months	169	53.1	124	91.9	250	95.4	543	75.9
Live within 10-min walk of cave/mine with bats inside	1	0.3	8	5.9	56	21.4	65	9.1
Been in cave/mine with bats inside in last 4 months	1	0.3	8	5.9	46	17.6	55	7.7
Been in building with bats inside in last 4 months	58	18.2	31	23.0	78	29.8	167	23.4
Bats come inside home	34	10.7	12	8.9	57	21.8	103	14.4
Fruit trees on compound that bats feed on	59	18.6	29	21.5	103	39.3	191	26.7
Eaten fruit with bite marks	90	28.3	60	44.4	120	45.8	270	37.8
Raised/cared for bats	1	0.3	0	0.0	0	0.0	1	0.1
Hunted or trapped bats	1	0.3	2	1.5	9	3.4	12	1.7
Slaughtered bats	0	0.0	1	0.7	4	1.5	5	0.7
Injured while butchering/slaughtering bat	0	0.0	0	0.0	0	0.0	0	0.0
Cooked bats	1	0.3	1	0.7	7	2.7	9	1.3
Eaten raw/undercooked bats	0	0.0	0	0.0	2	0.8	2	0.3
Scratched/bitten by bats	0	0.0	0	0.0	1	0.4	1	0.1
Touched dead bat	0	0.0	1	0.7	4	1.5	5	0.7

*Note:* Percentages calculated by site total.

⁎ Select all that apply.

### 3.5 Multivariable logistic regression

**Fig. 2:**
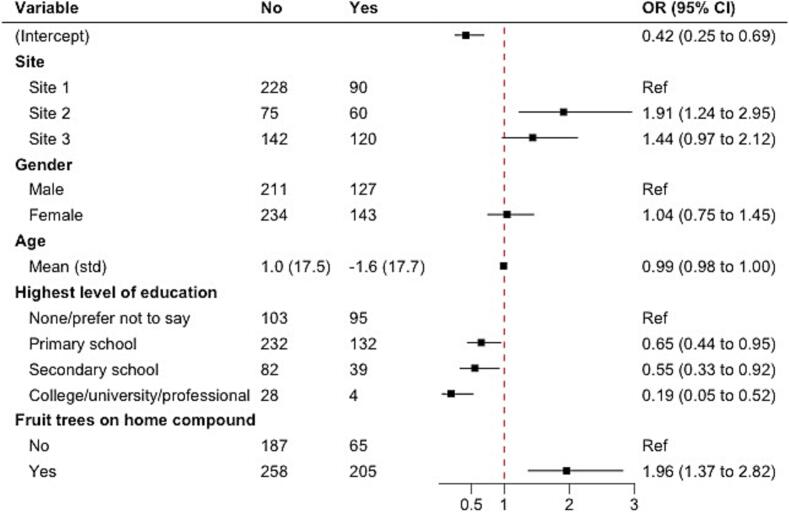
Multiple logistic regression of having eaten fruit bearing bite marks, all sites (n = 715).

Compared to Site 1, Site 2 residents were significantly more likely (OR 1.91, CI 1.24–2.95) to eat fruit bearing bite marks. Residents in Site 3 were also predicted to have higher odds, though the 95 % CI minimally overlapped one (OR 1.44, CI 0.97–2.12). Higher levels of education were negatively associated with eating fruit bearing bite marks. Compared to those who reported no education, those reporting secondary school or college/university/professional had 45 % and 81 % lower odds of eating fruit bearing bite marks, respectively (CIs 0.33–0.92 and 0.05–0.52). Participants with fruit trees on their home compound had almost twice the odds of eating fruit bearing bite marks, compared to those with no fruit trees (OR 1.96, CI 1.37–2.82). No statistically significant associations were observed for gender or age (Fig. 2).

**Fig. 3:**
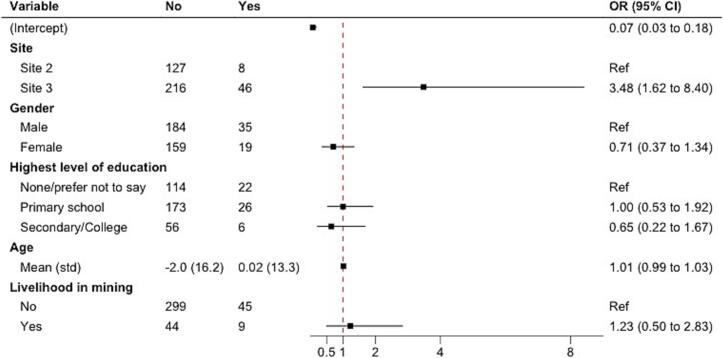
Multiple logistic regression of exposure to bats in a cave/mine in previous 4 months, sites 2 and 3, Western Region (*n* = 397).

For recent bat exposure in caves or mines, which was limited to Sites 2 and 3, participants at Site 3 had 3.48 higher odds (CI 1.62–8.40) of exposure to bats in caves or mines compared to Site 2. No statistically significant differences were observed for the other variables in the model, included working in mining (Fig. 3).

### 3.6 Results dissemination

Following completion of the final report, we presented our findings to the MoH and local authorities at the study sites. Discussions were had about increasing MVD surveillance, including serological surveys, and implementing methods to reduce bat contact.

## 4 Discussion

Our study provides evidence that within the populations involved in a Marburg virus disease outbreak, people had exposure to several potential spillover routes for Marburg or other zoonotic viruses carried by bats. Participants across all sites reported eating fruit bearing bite marks, which could be a route of exposure to pathogens excreted in bat saliva and other excreta [13,[24], [25], [26], [27]]. Entering caves or mines is a known risk factor for exposure to Marburg virus and nearly 20 % of participants in Site 2 reported recent exposure to bats in caves or mines [5,12,28].

In all logistic regression models, site was a significant predictor of potential ERB exposure, suggesting that exposure levels vary by location based on factors not measured by this study, such as land use and bat movement. When examining risk factors associated with eating fruit bearing bite marks, higher levels of education were found to be protective, while having fruit trees present on the participant's home compound increased the odds of this potential ERB exposure. This suggests that continued education efforts could be an effective tool to prevent exposures that may lead to spillover of MARV and other zoonotic diseases.

Site 3 was shown to have the highest odds of exposure to bats inside a cave or mine. Of note, participants who reported working in mining did not have higher odds of exposure to bats in caves or mines than non-miners. This may be due to bats being less likely to roost in active mines, frequent community exposure to caves and mines regardless of profession, or misclassification bias, if mining is underreported because most mines in the area operate illegally.

Bats inside buildings were commonly reported across all sites; while ERBs don't typically roost in occupied buildings, other bats inside houses and frequently used buildings may present a risk of exposure to other zoonotic pathogens, such as rabies virus. Few participants reported hunting, preparing, or eating bats, although bat hunting and consumption have been more commonly reported in previous studies in Ghana [[29], [30], [31]]. The frequency of these activities may be underreported due to stigma, or decreasing due to changing risk perceptions [32]. Pigs are susceptible to Ebola viruses and are considered a potential amplifier host for Marburg virus [33]. We included contact with pigs in our questionnaire to capture information about other potential sources of filovirus exposure [34,35]. While swine contact was not common at any sites, it could increase due to a government program encouraging pig farming and should be monitored as a potential spillover route.

One Site 3 participant reported fever with unexplained bleeding or bruising, fever with severe headache and fatigue, and fever with severe nausea, vomiting, or diarrhea within the previous four months. While these symptoms could have many causes, unexplained bleeding or bruising is indicative of VHFs such as MVD. However, it is unknown whether those symptoms occurred simultaneously or during distinct illness episodes.

To date, no serological surveys have been conducted in these communities. We recommend this as a next step to help identify potential unreported cases linked to this outbreak or other instances of exposure to MARV or other filoviruses. If the index case was infected in one of these sites, it is plausible that other filovirus spillover events have occurred but gone unnoticed due to lack of surveillance. Strengthening community surveillance systems, along with educational outreach targeting high-risk exposures, could help reduce the risk of MARV spillover and spread.

Our team noted that crop farming and, in the area near Sites 2 and 3, small-scale mining had caused environmental disruption. Land use change is one of the most significant drivers of disease spillover, disrupting natural ecosystems and bringing humans, domestic animals, and wild animals into closer contact [35]. Ebola outbreaks in Central and West Africa have been correlated with deforestation, and monitoring areas with high rates of land use change could be incorporated into zoonotic disease surveillance and mitigation strategies [36,37].

### 4.1 Limitations

While we focused this study on behaviors associated with spillover, it is possible that the first known case was infected by another person, and that spillover occurred at a different location outside our survey area. Another limitation of this study is that households were selected using haphazard sampling, and the samples may not be fully representative of the communities. Additionally, due to time and resource constraints, we were not able to achieve our target sample sizes for Site 2 and 3, limiting our power to describe those sites. The accuracy of self-reported information may also be limited by recall and social desirability bias.

## 5 Conclusions

This study has provided useful baseline data characterizing potential exposures to MARV hosts through pathways such as eating bitten fruit and entering caves/mines where bats are present. Future studies can build upon this preliminary data with serological surveys of humans and domestic and wild animals. Increased community and clinic-based surveillance could help further characterize the risk and incidence of MVD and other VHFs. The 2024 MVD outbreak in Rwanda was linked to mining in a cave, further underscoring the importance of identifying communities with higher exposure risk due to mining activities. We recommend implementing educational and occupational risk-mitigation campaigns in these communities, emphasizing avoiding ERB habitats, not attempting to exterminate bats from mines (which has been shown to increase MARV transmission [38]), and wearing appropriate PPE during high-risk activities.

## Article summary line

A behavioral risk assessment in three rural communities in Ghana following a July 2022 cluster of Marburg virus disease cases identified opportunities for exposure to Marburg virus and other bat-borne zoonoses.

## Funding

Support for this work was provided by United Kingdom (UK) Official Development Assistance through the UK 10.13039/501100006631Animal and Plant Health Agency (APHA). We thank Prof. Anthony R. Fooks and Dr. Flavie Vial from APHA for their support.

## CRediT authorship contribution statement

**Richard Suu-Ire:** Writing – review & editing, Supervision, Resources, Project administration, Methodology, Investigation, Funding acquisition, Data curation, Conceptualization. **Shannon Ball:** Writing – review & editing, Writing – original draft, Visualization, Validation, Software, Project administration, Methodology, Formal analysis, Data curation, Conceptualization. **Meyir Yiryele Ziekah:** Writing – review & editing, Supervision, Resources, Project administration, Investigation, Data curation. **Jean DeMarco:** Writing – review & editing, Supervision, Project administration, Methodology, Data curation, Conceptualization. **Morgan Kain:** Writing – review & editing, Visualization, Methodology, Formal analysis. **Amos Sarpong Agyei:** Writing – review & editing, Visualization, Resources, Project administration, Investigation, Data curation. **Jonathan H. Epstein:** Writing – review & editing, Supervision, Resources, Project administration, Methodology, Funding acquisition, Conceptualization.

## Declaration of competing interest

The authors declare that they have no known competing financial interests or personal relationships that could have appeared to influence the work reported in this paper.

## Data Availability

Data will be made available on request.

## Appendix A Supplementary data

Supplementary material 1 Image 1

Supplementary material 2 Image 2
