# Supplementary material for: Behavioral risk assessment of exposure to wild and domestic animals in response to a Marburg virus disease outbreak, Ghana 2022
Source: One Health. 2025 Mar 14;20:101010. doi: 10.1016/j.onehlt.2025.101010 (PMC11987680; doi:10.1016/j.onehlt.2025.101010)
Supplement: Supplementary file 1 — Supplementary material 1 [file mmc1.docx]

| **Supplement 1. Household characteristics** | | | | | | | | |
| --- | --- | --- | --- | --- | --- | --- | --- | --- |
|  | **Ashanti Region** | | **Western Region** | | | |  | |
|  | **Site 1 (n=318)** | | **Site 2 (n=135)** | | **Site 3 (n=262)** | | **Total (n=715)** | |
| **Variable** | **n** | **%** | **n** | **%** | **n** | **%** | **n** | **%** |
| **Number living in household** | | | | | | | | |
| 1-5 | 189 | 59.4 | 97 | 71.9 | 163 | 62.2 | 449 | 62.8 |
| 6-10 | 122 | 38.4 | 35 | 25.9 | 85 | 32.4 | 242 | 33.8 |
| >10 | 7 | 2.2 | 3 | 2.2 | 14 | 5.3 | 24 | 3.4 |
| **Food storage^*^** | | | | | | | | |
| No container | 113 | 35.5 | 69 | 51.1 | 109 | 41.6 | 291 | 40.7 |
| Covered, sealed container | 295 | 92.8 | 111 | 82.2 | 249 | 95.0 | 655 | 91.6 |
| Covered, unsealed container | 251 | 78.9 | 104 | 77.0 | 223 | 85.1 | 578 | 80.8 |
| Uncovered container | 124 | 39.0 | 63 | 46.7 | 142 | 54.2 | 329 | 46.0 |
| Don't know/prefer not to say | 2 | 0.6 | 3 | 2.2 | 1 | 0.4 | 6 | 0.8 |
| **Drinking water source^*^** | | | | | | | | |
| Indoor pipe | 29 | 9.1 | 0 | 0.0 | 1 | 0.4 | 30 | 4.2 |
| Outdoor pipe | 54 | 17.0 | 76 | 56.3 | 62 | 23.7 | 192 | 26.9 |
| Covered well | 24 | 7.5 | 45 | 33.3 | 141 | 53.8 | 210 | 29.4 |
| Uncovered well | 7 | 2.2 | 8 | 5.9 | 100 | 38.2 | 115 | 16.1 |
| Stream/river | 228 | 71.7 | 15 | 11.1 | 77 | 29.4 | 320 | 44.8 |
| Pond/lake | 1 | 0.3 | 0 | 0.0 | 7 | 2.7 | 8 | 1.1 |
| Purchased bottles/sachets | 100 | 31.4 | 26 | 19.3 | 2 | 0.8 | 128 | 17.9 |
| Other | 6 | 1.9 | 6 | 4.4 | 31 | 11.8 | 43 | 6.0 |
| Don't know/prefer not to say | 0 | 0.0 | 0 | 0.0 | 0 | 0.0 | 0 | 0.0 |
| **Water storage^*^** | | | | | | | | |
| Covered container | 275 | 86.5 | 125 | 92.6 | 249 | 95.0 | 649 | 90.8 |
| Uncovered container | 55 | 17.3 | 10 | 7.4 | 100 | 38.2 | 165 | 23.1 |
| No container | 18 | 5.7 | 5 | 3.7 | 2 | 0.8 | 25 | 3.5 |
| Don't know/prefer not to say | 1 | 0.3 | 3 | 2.2 | 0 | 0.0 | 4 | 0.6 |
| **Toilet facilities^*^** | | | | | | | | |
| Private indoor toilet | 98 | 30.8 | 1 | 0.7 | 2 | 0.8 | 101 | 14.1 |
| Private outdoor latrine | 110 | 34.6 | 83 | 61.5 | 223 | 85.1 | 416 | 58.2 |
| Public toilet | 114 | 35.8 | 51 | 37.8 | 33 | 12.6 | 198 | 27.7 |
| Bucket | 0 | 0.0 | 0 | 0.0 | 0 | 0.0 | 0 | 0.0 |
| Bush | 10 | 3.1 | 3 | 2.2 | 4 | 1.5 | 17 | 2.4 |
| Other | 0 | 0.0 | 0 | 0.0 | 3 | 1.1 | 3 | 0.4 |
| Don't know/prefer not to say | 0 | 0.0 | 0 | 0.0 | 0 | 0.0 | 0 | 0.0 |
| *Note:* Percentages calculated by site total. |  |  |  |  |  |  |  |  |
| ^*^Select all that apply. |  |  |  |  |  |  |  |  |

| **Supplement 2. History of severe illness and medical treatment seeking among all participants** | | | | | | | | |
| --- | --- | --- | --- | --- | --- | --- | --- | --- |
|  | **Ashanti Region** | | **Western Region** | | | |  | |
|  | **Site 1 (n=318)** | | **Site 2 (n=135)** | | **Site 3 (n=262)** | | **Total (n=715)** | |
| **Variable** | **n** | **%** | **n** | **%** | **n** | **%** | **n** | **%** |
| **Severe illness** | | | | | | | | |
| Severe illness in last 4 months | 52 | 16.4 | 21 | 15.6 | 38 | 14.5 | 111 | 15.5 |
| **Treatment for medical problems^*^** | | | | | | | | |
| Clinic | 65 | 20.4 | 22 | 16.3 | 80 | 30.5 | 167 | 23.4 |
| Hospital | 209 | 65.7 | 55 | 40.7 | 106 | 40.5 | 370 | 51.7 |
| Mobile clinic | 0 | 0.0 | 0 | 0.0 | 1 | 0.4 | 1 | 0.1 |
| Community health worker | 0 | 0.0 | 0 | 0.0 | 1 | 0.4 | 1 | 0.1 |
| Traditional healer/remedy | 30 | 9.4 | 43 | 31.9 | 72 | 27.5 | 145 | 20.3 |
| Dispensary or pharmacy | 69 | 21.7 | 63 | 46.7 | 97 | 37.0 | 229 | 32.0 |
| Self-medication | 100 | 31.4 | 82 | 60.7 | 166 | 63.4 | 348 | 48.7 |
| Do not usually treat | 11 | 3.5 | 3 | 2.2 | 1 | 0.4 | 15 | 2.1 |
| Other | 1 | 0.3 | 4 | 3.0 | 3 | 1.1 | 8 | 1.1 |
| *Note:* Percentages calculated by site total. |  |  |  |  |  |  |  |  |
| ^*^Select all that apply. |  |  |  |  |  |  |  |  |

| **Supplement 3. Symptoms and health beliefs among participants who self-reported severe illness within the prior 4 months** | | | | | | | | |
| --- | --- | --- | --- | --- | --- | --- | --- | --- |
|  | **Ashanti Region** | | **Western Region** | | | |  | |
|  | **Site 1 (n=52)** | | **Site 2 (n=21)** | | **Site 3 (n=38)** | | **Total (n=111)** | |
| **Variable** | **n** | **%** | **n** | **%** | **n** | **%** | **n** | **%** |
| **Symptoms^*^** | | | | | | | | |
| Fever | 41 | 78.8 | 14 | 66.7 | 30 | 78.9 | 85 | 11.9 |
| Fever with severe headache and fatigue | 29 | 55.8 | 12 | 57.1 | 28 | 73.7 | 69 | 9.7 |
| Fever with severe nausea, vomiting, or diarrhea | 16 | 30.8 | 3 | 14.3 | 9 | 23.7 | 28 | 3.9 |
| Fever with rash | 5 | 9.6 | 0 | 0.0 | 0 | 0.0 | 5 | 0.7 |
| Fever with bleeding or bruising not related to injury | 0 | 0.0 | 0 | 0.0 | 1 | 2.6 | 1 | 0.1 |
| Headache | 35 | 67.3 | 14 | 66.7 | 33 | 86.8 | 82 | 11.5 |
| Severe fatigue and/or weakness | 31 | 59.6 | 14 | 66.7 | 28 | 73.7 | 73 | 10.2 |
| Bleeding or bruising not related to injury | 0 | 0.0 | 0 | 0.0 | 1 | 2.6 | 1 | 0.1 |
| Muscle aches | 34 | 65.4 | 13 | 61.9 | 28 | 73.7 | 75 | 10.5 |
| Cough and shortness of breath or difficulty breathing | 9 | 17.3 | 6 | 28.6 | 8 | 21.1 | 23 | 3.2 |
| Sore throat | 5 | 9.6 | 2 | 9.5 | 4 | 10.5 | 11 | 1.5 |
| Rash | 4 | 7.7 | 2 | 9.5 | 1 | 2.6 | 7 | 1.0 |
| Persistent rash | 1 | 1.9 | 2 | 9.5 | 1 | 2.6 | 4 | 0.6 |
| Abdominal pain | 18 | 34.6 | 9 | 42.9 | 14 | 36.8 | 41 | 5.7 |
| Diarrhea | 6 | 11.5 | 2 | 9.5 | 10 | 26.3 | 18 | 2.5 |
| Nausea and/or vomiting | 17 | 32.7 | 2 | 9.5 | 7 | 18.4 | 26 | 3.6 |
| Sores on skin | 3 | 5.8 | 3 | 14.3 | 1 | 2.6 | 7 | 1.0 |
| **Believed cause of illness^*^** | | | | | | | | |
| Contact with sick people | 0 | 0.0 | 0 | 0.0 | 0 | 0.0 | 0 | 0.0 |
| Contact with wild animals | 0 | 0.0 | 0 | 0.0 | 0 | 0.0 | 0 | 0.0 |
| Contact with other animals | 0 | 0.0 | 0 | 0.0 | 0 | 0.0 | 0 | 0.0 |
| Contact with germs | 6 | 11.5 | 0 | 0.0 | 3 | 7.9 | 9 | 1.3 |
| Bad food or water | 1 | 1.9 | 2 | 9.5 | 2 | 5.3 | 5 | 0.7 |
| Bad spirits/witchcraft | 1 | 1.9 | 0 | 0.0 | 0 | 0.0 | 1 | 0.1 |
| Mosquitoes | 9 | 17.3 | 1 | 4.8 | 8 | 21.1 | 18 | 2.5 |
| Work | 7 | 13.5 | 4 | 19.0 | 7 | 18.4 | 18 | 2.5 |
| Wound or injury | 5 | 9.6 | 5 | 23.8 | 1 | 2.6 | 11 | 1.5 |
| Don't know | 17 | 32.7 | 9 | 42.9 | 15 | 39.5 | 41 | 5.7 |
| Other | 10 | 19.2 | 1 | 4.8 | 5 | 13.2 | 16 | 2.2 |
| **Household transmission** | | | | | | | | |
| Someone else in household had  same illness at same time | 11 | 21.2 | 6 | 28.6 | 14 | 36.8 | 31 | 4.3 |
| *Note:* Percentages calculated by site total. |  |  |  |  |  |  |  |  |
| ^*^Select all that apply. |  |  |  |  |  |  |  |  |

| **Supplement 4. Travel in prior 4 months** | | | | | | | | |
| --- | --- | --- | --- | --- | --- | --- | --- | --- |
|  | **Ashanti Region** | | **Western Region** | | | |  | |
|  | **Site 1 (n=318)** | | **Site 2 (n=135)** | | **Site 3 (n=262)** | | **Total (n=715)** | |
| **Variable** | **n** | **%** | **n** | **%** | **n** | **%** | **n** | **%** |
| **Travelled in last 4 months** | | | | | | | | |
| No | 142 | 44.7 | 90 | 66.7 | 174 | 66.4 | 406 | 56.8 |
| Yes | 176 | 55.3 | 45 | 33.3 | 88 | 33.6 | 309 | 43.2 |
| **Number of travel destinations** | | | | | | | | |
| 1 | 126 | 39.6 | 43 | 31.9 | 81 | 30.9 | 250 | 35.0 |
| 2 | 36 | 11.3 | 2 | 1.5 | 5 | 1.9 | 43 | 6.0 |
| 3 | 7 | 2.2 | 0 | 0.0 | 1 | 0.4 | 8 | 1.1 |
| 4 | 1 | 0.3 | 0 | 0.0 | 0 | 0.0 | 1 | 0.1 |
| 5 | 1 | 0.3 | 0 | 0.0 | 0 | 0.0 | 1 | 0.1 |
| N/A | 147 | 46.2 | 90 | 66.7 | 175 | 66.8 | 412 | 57.6 |
| *Note:* Percentages calculated by site total. |  |  |  |  |  |  |  |  |

| **Supplement 5. Travel destinations in previous 4 months** | | | | | | | | |
| --- | --- | --- | --- | --- | --- | --- | --- | --- |
|  | **Ashanti Region** | | **Western Region** | | | |  | |
|  | **Site 1 (n=234)** | | **Site 2 (n=47)** | | **Site 3 (n=95)** | | **Total (n=376)** | |
| **Variable** | **n** | **%** | **n** | **%** | **n** | **%** | **n** | **%** |
| **Countries outside Ghana** | | | | | | | | |
| Côte d'Ivoire | 2 | 0.9 | 0 | 0.0 | 1 | 1.1 | 3 | 0.4 |
| Togo | 0 | 0.0 | 2 | 4.3 | 0 | 0.0 | 2 | 0.3 |
| **Regions within Ghana** | | | | | | | | |
| Ahafo | 1 | 0.4 | 1 | 2.1 | 5 | 5.3 | 7 | 1.0 |
| Ashanti | 155 | 66.2 | 16 | 34 | 22 | 23.2 | 193 | 27.0 |
| Bono | 2 | 0.9 | 2 | 4.3 | 7 | 7.4 | 11 | 1.5 |
| Bono East | 3 | 1.3 | 1 | 2.1 | 5 | 5.3 | 9 | 1.3 |
| Central | 12 | 5.1 | 6 | 12.8 | 1 | 1.1 | 19 | 2.7 |
| Eastern | 4 | 1.7 | 0 | 0.0 | 2 | 2.1 | 6 | 0.8 |
| Greater Accra | 21 | 9 | 4 | 8.5 | 1 | 1.1 | 26 | 3.6 |
| Northern East | 0 | 0.0 | 0 | 0.0 | 1 | 1.1 | 1 | 0.1 |
| Savannah | 2 | 0.9 | 0 | 0.0 | 11 | 11.6 | 13 | 1.8 |
| Upper East | 1 | 0.4 | 1 | 2.1 | 4 | 4.2 | 6 | 0.8 |
| Upper West | 1 | 0.4 | 5 | 10.6 | 19 | 20 | 25 | 3.5 |
| Volta | 2 | 0.9 | 0 | 0.0 | 1 | 1.1 | 3 | 0.4 |
| Western | 12 | 5.1 | 6 | 12.8 | 13 | 13.7 | 31 | 4.3 |
| Western North | 4 | 1.7 | 3 | 6.4 | 1 | 1.1 | 8 | 1.1 |
| *Note:* Percentages calculated by total number of trips per site. | | |  |  |  |  |  |  |

| **Supplement 6. Reasons for travel in previous 4 months** | | | | | | | | |
| --- | --- | --- | --- | --- | --- | --- | --- | --- |
|  | **Ashanti Region** | | **Western Region** | | | |  | |
|  | **Site 1 (n=234)** | | **Site 2 (n=47)** | | **Site 3 (n=95)** | | **Total (n=376)** | |
| **Variable** | **n** | **%** | **n** | **%** | **n** | **%** | **n** | **%** |
| **Work** | | | | | | | | |
| No | 185 | 79.1 | 42 | 89.4 | 86 | 90.5 | 313 | 43.8 |
| Yes | 44 | 18.8 | 5 | 10.6 | 8 | 8.4 | 57 | 8.0 |
| Prefer not to say | 5 | 2.1 | 0 | 0.0 | 1 | 1.1 | 6 | 0.8 |
| **Visiting family** | | | | | | | | |
| No | 129 | 55.1 | 16 | 34 | 28 | 29.5 | 173 | 24.2 |
| Yes | 100 | 42.7 | 31 | 66 | 66 | 69.5 | 197 | 27.6 |
| Prefer not to say | 5 | 2.1 | 0 | 0.0 | 1 | 1.1 | 6 | 0.8 |
| **Moving** | | | | | | | | |
| No | 229 | 97.9 | 47 | 100 | 93 | 97.9 | 369 | 51.6 |
| Yes | 0 | 0 | 0 | 0 | 1 | 1.1 | 1 | 0.1 |
| Prefer not to say | 5 | 2.1 | 0 | 0.0 | 1 | 1.1 | 6 | 0.8 |
| **Religious reasons** | | | | | | | | |
| No | 226 | 96.6 | 47 | 100 | 92 | 96.8 | 365 | 51.0 |
| Yes | 3 | 1.3 | 0 | 0 | 2 | 2.1 | 5 | 0.7 |
| Prefer not to say | 5 | 2.1 | 0 | 0.0 | 1 | 1.1 | 6 | 0.8 |
| **Holiday/vacation** | | | | | | | | |
| No | 226 | 96.6 | 47 | 100 | 94 | 98.9 | 367 | 51.3 |
| Yes | 3 | 1.3 | 0 | 0 | 0 | 0 | 3 | 0.4 |
| Prefer not to say | 5 | 2.1 | 0 | 0.0 | 1 | 1.1 | 6 | 0.8 |
| **Going to hospital** | | | | | | | | |
| No | 221 | 94.4 | 47 | 100 | 89 | 93.7 | 357 | 49.9 |
| Yes | 8 | 3.4 | 0 | 0 | 5 | 5.3 | 13 | 1.8 |
| Prefer not to say | 5 | 2.1 | 0 | 0.0 | 1 | 1.1 | 6 | 0.8 |
| **Going to market** | | | | | | | | |
| No | 186 | 79.5 | 46 | 97.9 | 91 | 95.8 | 323 | 45.2 |
| Yes | 43 | 18.4 | 1 | 2.1 | 3 | 3.2 | 47 | 6.6 |
| Prefer not to say | 5 | 2.1 | 0 | 0.0 | 1 | 1.1 | 6 | 0.8 |
| **Other** | | | | | | | | |
| No | 188 | 80.3 | 38 | 80.9 | 77 | 81.1 | 303 | 42.4 |
| Yes | 41 | 17.5 | 9 | 19.1 | 17 | 17.9 | 67 | 9.4 |
| Prefer not to say | 5 | 2.1 | 0 | 0.0 | 1 | 1.1 | 6 | 0.8 |
| **Don't know/prefer not to say** | | | | | | | | |
| No | 228 | 97.4 | 46 | 97.9 | 94 | 98.9 | 368 | 51.5 |
| Yes | 1 | 0.4 | 1 | 2.1 | 0 | 0 | 2 | 0.3 |
| Prefer not to say | 5 | 2.1 | 0 | 0.0 | 1 | 1.1 | 6 | 0.8 |
| *Note:* Percentages calculated by total number of trips per site. Six participants traveled but preferred not to share travel reasons. | | | | | | | | |

| **Supplement 7. Frequency of visits to forest areas** | | | | | | | | |
| --- | --- | --- | --- | --- | --- | --- | --- | --- |
|  | **Ashanti Region** | | **Western Region** | | | |  | |
|  | **Site 1 (n=318)** | | **Site 2 (n=135)** | | **Site 3 (n=262)** | | **Total (n=715)** | |
| **Variable** | **n** | **%** | **n** | **%** | **n** | **%** | **n** | **%** |
| **Frequency of visits to forest areas** | | | | | | | | |
| 4 or more times a week | 117 | 36.8 | 84 | 62.2 | 209 | 79.8 | 410 | 57.3 |
| 1-3 times a week | 72 | 22.6 | 16 | 11.9 | 24 | 9.2 | 112 | 15.7 |
| 1-3 times a month | 33 | 10.4 | 8 | 5.9 | 15 | 5.7 | 56 | 7.8 |
| Less than once a month | 25 | 7.9 | 13 | 9.6 | 6 | 2.3 | 44 | 6.2 |
| Never | 71 | 22.3 | 14 | 10.4 | 7 | 2.7 | 92 | 12.9 |
| *Note:* |  |  |  |  |  |  |  |  |
| Percentages calculated by site total. |  |  |  |  |  |  |  |  |

| **Supplement 8. Reasons for visits to forest areas** | | | | | | | | |
| --- | --- | --- | --- | --- | --- | --- | --- | --- |
|  | **Ashanti Region** | | **Western Region** | | | |  | |
|  | **Site 1 (n=247)** | | **Site 2 (n=121)** | | **Site 3 (n=255)** | | **Total (n=715)** | |
| **R**easons for visits to forest areas | **n** | **%** | **n** | **%** | **n** | **%** | **n** | **%** |
| **Hunting** | | | | | | | | |
| No | 233 | 94.3 | 95 | 78.5 | 199 | 78.3 | 527 | 73.7 |
| Yes | 14 | 5.7 | 26 | 21.5 | 55 | 21.7 | 95 | 13.3 |
| **Collecting water** | | | | | | | | |
| No | 181 | 73.3 | 112 | 92.6 | 196 | 77.2 | 489 | 68.4 |
| Yes | 66 | 26.7 | 9 | 7.4 | 58 | 22.8 | 133 | 18.6 |
| **Gathering fruit/vegetables** | | | | | | | | |
| No | 170 | 68.8 | 82 | 67.8 | 188 | 74 | 440 | 61.5 |
| Yes | 77 | 31.2 | 39 | 32.2 | 66 | 26 | 182 | 25.5 |
| **Collecting firewood** | | | | | | | | |
| No | 141 | 57.1 | 63 | 52.1 | 144 | 56.7 | 348 | 48.7 |
| Yes | 106 | 42.9 | 58 | 47.9 | 110 | 43.3 | 274 | 38.3 |
| **Fishing** | | | | | | | | |
| No | 246 | 99.6 | 120 | 99.2 | 253 | 99.6 | 619 | 86.6 |
| Yes | 1 | 0.4 | 1 | 0.8 | 1 | 0.4 | 3 | 0.4 |
| **Other** | | | | | | | | |
| No | 147 | 59.5 | 59 | 48.8 | 133 | 52.4 | 339 | 47.4 |
| Yes | 100 | 40.5 | 62 | 51.2 | 121 | 47.6 | 283 | 39.6 |
| **Don't know/prefer not to say** | | | | | | | | |
| No | 244 | 98.8 | 121 | 100 | 251 | 98.8 | 616 | 86.2 |
| Yes | 3 | 1.2 | 0 | 0 | 3 | 1.2 | 6 | 0.8 |
| *Note:* Percentages calculated by site total. |  |  |  |  |  |  |  |  |

| **Supplement 9. Animal contact in previous 4 months** | | | | | | | | |
| --- | --- | --- | --- | --- | --- | --- | --- | --- |
|  | **Ashanti Region** | | **Western Region** | | | |  | |
|  | **Site 1 (n=318)** | | **Site 2 (n=135)** | | **Site 3 (n=262)** | | **Total (n=715)** | |
|  | **n** | **%** | **n** | **%** | **n** | **%** | **n** | **%** |
| **Animal taxa^*^** | | | | | | | | |
| Rodent/shrew | 115 | 36.2 | 95 | 70.4 | 184 | 70.2 | 394 | 55.1 |
| Bat | 35 | 11.0 | 15 | 11.1 | 67 | 25.6 | 117 | 16.4 |
| Monkey/chimp | 3 | 0.9 | 2 | 1.5 | 0 | 0.0 | 5 | 0.7 |
| Wild ungulate | 17 | 5.3 | 14 | 10.4 | 26 | 9.9 | 57 | 8.0 |
| Goats/sheep | 110 | 34.6 | 16 | 11.9 | 88 | 33.6 | 214 | 29.9 |
| Swine | 4 | 1.3 | 1 | 0.7 | 4 | 1.5 | 9 | 1.3 |
| Cattle/buffalo | 46 | 14.5 | 2 | 1.5 | 3 | 1.1 | 51 | 7.1 |
| Dog | 128 | 40.3 | 54 | 40.0 | 143 | 54.6 | 325 | 45.5 |
| Cat | 183 | 57.5 | 56 | 41.5 | 159 | 60.7 | 398 | 55.7 |
| Other | 142 | 44.7 | 76 | 56.3 | 200 | 76.3 | 418 | 58.5 |
| **Contact type^*^** | | | | | | | | |
| Raised/cared for | 176 | 55.3 | 88 | 65.2 | 238 | 90.8 | 502 | 70.2 |
| Come inside home | 197 | 61.9 | 95 | 70.4 | 184 | 70.2 | 476 | 66.6 |
| Hunted/trapped | 32 | 10.1 | 25 | 18.5 | 62 | 23.7 | 119 | 16.6 |
| Slaughtered | 27 | 8.5 | 22 | 16.3 | 75 | 28.6 | 124 | 17.3 |
| Cooked or handled meat/organs/blood | 94 | 29.6 | 47 | 34.8 | 100 | 38.2 | 241 | 33.7 |
| Injured while slaughtering/butchering | 15 | 4.7 | 5 | 3.7 | 15 | 5.7 | 35 | 4.9 |
| Eaten raw or smoked meat/organs/blood | 64 | 20.1 | 28 | 20.7 | 99 | 37.8 | 191 | 26.7 |
| Scratched/bitten by | 7 | 2.2 | 5 | 3.7 | 6 | 2.3 | 18 | 2.5 |
| Touched dead animal | 35 | 11.0 | 6 | 4.4 | 27 | 10.3 | 68 | 9.5 |
| *Note:* Percentages calculated by site total. |  |  |  |  |  |  |  |  |
| ^*^Select all that apply. |  |  |  |  |  |  |  |  |

**Supplement 10. Heatmap of animal contact in previous 4 months**


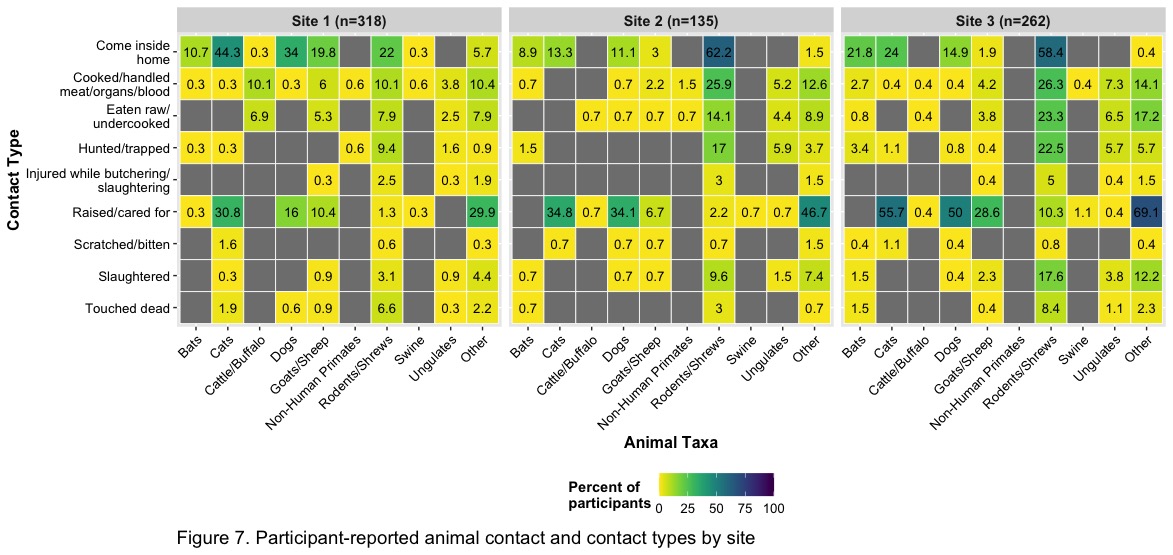


| **Supplement 11. Other animal exposures and behaviors** | | | | | | | | |
| --- | --- | --- | --- | --- | --- | --- | --- | --- |
|  | **Ashanti Region** | | **Western Region** | | | |  | |
|  | **Site 1 (n=318)** | | **Site 2 (n=135)** | | **Site 3 (n=262)** | | **Total (n=715)** | |
| **Exposure type** | **n** | **%** | **n** | **%** | **n** | **%** | **n** | **%** |
| **Fruit trees on compound** | | | | | | | | |
| No | 155 | 48.7 | 47 | 34.8 | 45 | 17.2 | 247 | 34.5 |
| Yes | 163 | 51.3 | 84 | 62.2 | 216 | 82.4 | 463 | 64.8 |
| Don't Know | 0 | 0.0 | 4 | 3.0 | 1 | 0.4 | 5 | 0.7 |
| **Do livestock eat fruit that has fallen on ground** | | | | | | | | |
| No | 85 | 26.7 | 15 | 11.1 | 63 | 24.0 | 163 | 22.8 |
| Yes | 19 | 6.0 | 6 | 4.4 | 56 | 21.4 | 81 | 11.3 |
| Don't Know | 72 | 22.6 | 54 | 40.0 | 90 | 34.4 | 216 | 30.2 |
| N/A | 142 | 44.7 | 60 | 44.4 | 53 | 20.2 | 255 | 35.7 |
| **Possible to buy bushmeat in home community** | | | | | | | | |
| No | 62 | 19.5 | 27 | 20.0 | 84 | 32.1 | 173 | 24.2 |
| Yes | 162 | 50.9 | 65 | 48.1 | 122 | 46.6 | 349 | 48.8 |
| Don't Know | 94 | 29.6 | 43 | 31.9 | 56 | 21.4 | 193 | 27.0 |
| **Believe that animals can give a disease to a human** | | | | | | | | |
| No | 8 | 2.5 | 7 | 5.2 | 15 | 5.7 | 30 | 4.2 |
| Yes | 291 | 91.5 | 119 | 88.1 | 231 | 88.2 | 641 | 89.7 |
| Don't Know | 19 | 6.0 | 9 | 6.7 | 16 | 6.1 | 44 | 6.2 |
| **Seen group of dead animals in last 4 months** | | | | | | | | |
| No | 307 | 96.5 | 130 | 96.3 | 256 | 97.7 | 693 | 96.9 |
| Yes | 11 | 3.5 | 5 | 3.7 | 5 | 1.9 | 21 | 2.9 |
| Don't Know | 0 | 0.0 | 0 | 0.0 | 1 | 0.4 | 1 | 0.1 |
| **What done with fruit that has bite marks^*^** | | | | | | | | |
| Avoid | 19 | 6.0 | 5 | 3.7 | 2 | 0.8 | 26 | 3.6 |
| Clean then eat | 48 | 15.1 | 28 | 20.7 | 56 | 21.4 | 132 | 18.5 |
| Cut off area with bite marks then eat | 59 | 18.6 | 53 | 39.3 | 87 | 33.2 | 199 | 27.8 |
| Just eat | 9 | 2.8 | 6 | 4.4 | 29 | 11.1 | 44 | 6.2 |
| Throw away | 188 | 59.1 | 81 | 60.0 | 150 | 57.3 | 419 | 58.6 |
| Other | 2 | 0.6 | 0 | 0.0 | 0 | 0.0 | 2 | 0.3 |
| N/A | 34 | 10.7 | 3 | 2.2 | 11 | 4.2 | 48 | 6.7 |
| **What done with animal found dead^*^** | | | | | | | | |
| Avoid it | 15 | 4.7 | 3 | 2.2 | 13 | 5.0 | 31 | 4.3 |
| Dispose of/bury it | 12 | 3.8 | 2 | 1.5 | 12 | 4.6 | 26 | 3.6 |
| Eat | 8 | 2.5 | 1 | 0.7 | 3 | 1.1 | 12 | 1.7 |
| Sell | 0 | 0.0 | 0 | 0.0 | 1 | 0.4 | 1 | 0.1 |
| Share | 0 | 0.0 | 0 | 0.0 | 0 | 0.0 | 0 | 0.0 |
| Feed to domestic animals | 0 | 0.0 | 0 | 0.0 | 1 | 0.4 | 1 | 0.1 |
| Other | 1 | 0.3 | 0 | 0.0 | 2 | 0.8 | 3 | 0.4 |
| N/A | 283 | 89.0 | 129 | 95.6 | 235 | 89.7 | 647 | 90.5 |
| *Note:* Percentages calculated by site total. |  |  |  |  |  |  |  |  |
| ^*^Select all that apply. |  |  |  |  |  |  |  |  |
